# Genital self-mutilation in erectile disorder

**DOI:** 10.4103/0019-5545.31623

**Published:** 2006

**Authors:** C.Y. Sudarshan, K. Nagaraja Rao, S.V. Santosh

**Affiliations:** * Professor of Psychiatry, Department of Psychiatry, J.J.M. Medical College, Davangere 577004, Karnataka; ** Professor of Psychiatry, Department of Psychiatry, J.J.M. Medical College, Davangere 577004, Karnataka; *** Postgraduate student, Department of Psychiatry, J.J.M. Medical College, Davangere 577004, Karnataka

**Keywords:** Genital self-mutilation, erectile disorder

## Abstract

The majority of cases of genital self-mutilation reported in the literature have been in patients with psychosis. We report an unusual case of genital self-mutilation in erectile disorder. It is suggested that genital self-mutilation may be a pathway out of diverse psychological disorders and in non-psychotic cases it could be an expression of a psychotic solution to a conflict and may be influenced by cultural factors.

## INTRODUCTION

Genital self-mutilation is a severe form of self-injurious behaviour. A review of the literature suggests that it is usually associated with psychotic illness. Greilsheimer and Groves,^1^ in a group of 52 cases of genital self-mutilation, found 87% to be psychotic and 13% to be non-psychotic. The psychotic cases ranged from those with functional psychosis to those with brain damage. The non-psychotic cases included character disorders, transvestism and complex religious or cultural beliefs. Aboseif *et al.*^2^ in a series of 14 patients of self-inflicted genital injuries, found 65% of cases to be psychotic and 35% to be non-psychotic. There have been sporadic cases of non-psychotic genital self-mutilation in the literature.^3^–^5^ Various forms of psychopathology have been postulated in such cases. We report penile self-mutilation in a case of erectile disorder. In this case genital mutilation was a psychotic solution to a conflict and was influenced by cultural factors.

## THE CASE

A 29-year-old Hindu male reported to the emergency department after having chopped off his penis at its base with a chisel. The wound was sutured by a surgeon after haemo-stasis. Five days later he was referred for psychiatric evaluation.

The patient reported feelings of sadness since the past 7 months, whenever his mother and relatives asked him to get married. He had difficulty in initiating sleep and had decreased appetite whenever he thought of marriage. He stated that he did not want to get married as most of the marriages among his friends and relatives including that of his brother had ended up with the couples separating from their parents. He feared that his marriage would also result in separation from his mother who was a widow, and he would be failing in his duty to take care of her during her old age. When the pressure mounted on the patient to get married, he thought that if he cut off his penis, he would not need to get married and could live with his mother forever. He had made a futile attempt at penile amputation 15 days ago, but succeeded this time. On repeated probing it was found that he had poor erection while masturbating and while watching pornographic material since his adolescence. He had consulted a sexologist for this complaint, who had suggested an operation. He did not resort to any treatment fearing the high cost involved. He did not confide in any one fearing shame and stigma. He was doubtful of his sexual adequacy if married, and was scared of the consequences of marriage in such a situation, in addition to separation from his mother.

He was last of three siblings. His older brother and sister were married and were living separately. His father had died when he was 12 years old. He was educated up to XII standard. All his education was in institutions meant only for boys. He had a feeling of dislike towards women since childhood and communicated poorly with female relatives and other women. He reported nocturnal emissions and had masturbated occasionally without any fantasies. He was a taxi driver and was earning adequately to maintain a family.

History did not reveal any delusions, hallucinations and obsessions. He had no doubts about his gender identity. He had no preference for feminine dress, play or role. He had no homosexual feelings. He had had no heterosexual experiences.

Mental status examination revealed no remorse about his action. He was not depressed either subjectively or objectively. He reported that he was relieved that he need not get married. In addition, he was happy that there was no fear of separation from his mother.

The patient was diagnosed to have erectile disorder as per DSM-IV. After a year of follow-up, the patient reported no psychological disorder. He was cheerful and had no worries about his future life.

## DISCUSSION

Some proposed psychopothologies in genital self-mutilation in non-psychotic illnesses are: gender dysphoria,^3^ psychotic solution to conflicts about the male role, male identification, guilt for sexual offences,^6^ attempts at a crude sex change operation in the presence of trans-sexuality^3^,^5^ hypersexuality,^7^ a rational suicidal act,^4^ a means to get relieved from urinary symptoms.^8^ It has also been ascribed to sexual conflicts and offences,^9^ erotic purposes,^10^ body image preoccupation and distortion,^11^,^12^ expression of internalized frustration and aggression resulting in an impulsive suicide attempt.^13^,^14^

In the present case there was no evidence of gender dysphoria, conflict about the male role, male identification, guilt for sexual offences, trans-sexuality, hypersexuality, any distressing genito-urinary symptoms, frustration, aggression or suicidal intention. Though sadness, sleeplessness and decreased appetite were reported lasting over a period of 7 months, it was not reflected in the patient's behaviour and was ascribed to his having solved his marital conflict. Hence, it was possible that depression in this case was clinically subthreshold. The diagnosis of erectile disorder was entertained as there was history of inadequate erection in situations of sexual arousal. However, there was no actual heterosexual or homosexual activity involved.

It is suggested that there is no difference in the severity of self-inflicted injury between psychotic and non-psychotic groups of patients,^2^ and sometimes it could be a rational suicidal act.^4^ This was evident in the present case where he inflicted such a serious injury without seeking help.

Most cases of genital self-mutilation reported in the literature have been in psychotic patients with either functional or organic brain disease. This may be the result of selective reporting.^4^ This is an unusual case of genital self-mutilation in erectile disorder. There was no actual sexual contact and the person had not resorted to social support or alternative medical help, as is expected in such cases. The probable high cost of treatment and imagined possibility of shame and stigma precluded him from seeking such a solution to the problem. Thus, in this case, genital mutilation was a psychotic solution to a conflict on the individual plane and was influenced by cultural factors on the social plane.

In summary, genital self-mutilation may be a pathway out of diverse psychological disorders or behaviour and may be influenced by cultural factors. In non-psychotic cases it may be an expression of a psychotic solution to a conflict. It is suggested that genital self-mutilation in this case was a ‘psychotic solution’ because the action was extreme, disproportionate to the problem and unrealistic. He could have used less severe, realistic, even a ‘neurotic solution’ explained above to solve his problem.
